# Emergence of *bla*_*NDM−1*_ in *Pseudomonas aeruginosa* ST308 and ST773 from bloodstream infections in Egypt

**DOI:** 10.1186/s12866-026-05178-2

**Published:** 2026-06-22

**Authors:** Asmaa AbdulHak, Hamdallah H. Zedan, Hadir A. El-Mahallawy, Ahmed A Sayed, Mai M. Zafer

**Affiliations:** 1 Department of Microbiology and Immunology, Faculty of Pharmacy, Ahram Canadian University, Giza, Egypt; 2 Department of Microbiology and Immunology, Faculty of Pharmacy, Cairo University, Cairo, Egypt; 3 Department of Clinical Pathology, National Cancer Institute, Cairo University, Cairo, Egypt; 4 Genomics Program, Children’s cancer hospital Egypt 57357, Cairo, Egypt; 5 Department of Biochemistry, Faculty of Science, Ain Shams University, Cairo, Egypt

**Keywords:** *P.aeruginosa*, Carbapenem resistance, NDM-1, ST308, ST773, Integrative and conjugative element (ICE)

## Abstract

Carbapenem-resistant *Pseudomonas aeruginosa* (CRPA) remains a significant challenge to clinical management, particularly in critically ill hospitalized patients. The present study aimed to investigate the prevalence and molecular epidemiology of *bla*_NDM-1_ among 50 *Pseudomonas aeruginosa* isolates causing bloodstream infections in cancer patients in Egypt, and to characterize their antimicrobial resistance profiles, carbapenemase production, and biofilm-forming capacity. Antimicrobial susceptibility testing, minimum inhibitory concentrations, multiplex PCR for carbapenemase genes, biofilm assays, and multilocus sequence typing (MLST) were performed. Two representative isolates underwent whole-genome sequencing (WGS) to determine the genetic context of *bla*_*NDM-1*_. All isolates exhibited multidrug-resistant or extensively drug-resistant phenotypes. Carbapenem resistance was detected in 80% (*n* = 40) of isolates, and *bla*_*NDM-1*_was identified in 64% (*n* = 32), comprising 80% of carbapenem-resistant isolates. Strong biofilm formation was significantly associated with *bla*_*NDM-1*_ carriage (*P* = 0.0115). MLST analysis of a subset of *bla*_*NDM-1*_–producing isolates (*n* = 10) revealed two sequence types among *bla*_*NDM-1-*_producing isolates: ST308 (*n* = 6) and ST773 (*n* = 4). These findings indicate the dissemination of high-risk *Pseudomonas aeruginosa* clones ST308 and ST773 carrying *bla*_*NDM-1*_ in Egypt. WGS showed chromosomal integration of *bla*_*NDM-1*_within integrative and conjugative elements (ICEs) containing IS91-family transposases and multiple additional resistance genes. However, the relatively small sample size and the limited number of isolates subjected to WGS may restrict the generalizability of these findings. The presence of *bla*_*NDM-1*_ within mobile genetic platforms highlights the ongoing evolution and dissemination of CRPA in this clinical setting and calls for enhanced surveillance, as these lineages may act as reservoirs for the acquisition and dissemination of diverse antimicrobial resistance determinants via their putative ICE.

## Impact statement

Carbapenem-resistant *Pseudomonas aeruginosa* (CRPA) has been increasingly reported in Egypt, particularly from ICUs, oncology centers, and burn units. The predominant carbapenemase genes include *bla*_*VIM*_, *bla*_*IMP*_, and more recently *bla*_*NDM*_, highlighting its endemic presence and the need for ongoing molecular surveillance. This study suggests a possible local trend toward increased detection of *bla*_NDM−1_ (over other metallo-β-lactamases) among carbapenem-resistant *P. aeruginosa* (CRPA) isolates within this clinical setting. The detection of two high-risk clones, ST308 and ST773, as key drivers of *bla*_NDM−1_ transmission highlight the importance of enhanced surveillance, infection control measures, and antimicrobial stewardship to control the wide spread of these multidrug-resistant bacteria. The emergence of *P. aeruginosa* ST308 harboring *bla*_NDM−1_ in Egypt for the first time is particularly worrying, given its epidemic potential and ability to acquire additional resistance determinants through integrative and conjugative elements (ICEs). The findings of the present study highlight the significant role of horizontal gene transfer in the persistence and evolution of antimicrobial resistance, reinforcing the need for global efforts to contain the spread of *bla*_NDM−1_-producing *P. aeruginosa* and preserve the efficacy of available treatment options.

## Background

Carbapenem-resistant *Pseudomonas aeruginosa* (CRPA) poses a significant risk for healthcare-associated infections, especially among immunocompromised individuals. In Egypt it has emerged as a pressing public health concern [1, 2] The difficulty of combating carbapenem resistance is augmented by the potential for horizontal gene transfer of the carbapenemase-encoding genes [3]. Metallo-β-lactamases (MBLs) have one of the most powerful resistance mechanisms, being able to inactivate almost all β-lactam agents. Different types of MBLs have been described among *P. aeruginosa* isolates in Egypt, particularly VIM, IMP with different degrees of endemicity [4–6]. Recently, there has been a rising global prevalence of additional metallo-β-lactamases, including SPM, GIM and SIM, alongside an increasing occurrence of NDM producing strains, which are already endemic in *Enterobacterales* [7].

Recently, the emergence of *bla*_*NDM−1*_ in *P. aeruginosa* has raised particular concern due to its rapid global dissemination and its frequent association with mobile genetic elements, which facilitate horizontal gene transfer across different bacterial species. Although *bla*_*NDM−1*_ is well established in *Enterobacterales*, its increasing detection in *P. aeruginosa* highlights a significant epidemiological shift that warrants further investigation.

Notably, a recent study from our group [8] conducted in the same clinical setting reported a high prevalence of *bla*_*NDM−1*_ among *P. aeruginosa* clinical isolates. However, that study included isolates from multiple infection sites and did not investigate the genomic context of *bla*_*NDM−1*_. The present study builds upon these findings by focusing specifically on bloodstream infections and providing detailed genomic characterization, including analysis of integrative and conjugative elements (ICEs).

*P. aeruginosa* exhibits the capacity to form biofilms on various surfaces, including non-living surfaces and host epithelial cells [9]. The association between antimicrobial resistance and biofilm development is a significant issue in clinical settings, particularly concerning infections associated with biofilms [10].

Biofilm formation further complicates treatment, as it enhances bacterial survival and contributes to antimicrobial resistance through reduced antibiotic penetration and altered metabolic activity. The interplay between resistance determinants and biofilm formation remains insufficiently understood, particularly in clinical isolates.

Despite increasing reports of carbapenem-resistant *P. aeruginosa* in Egypt, data on the molecular epidemiology of *bla*_*NDM−1*_–producing strains, their clonal distribution, and genomic context remain limited.

In this context, the present study aimed to investigate the prevalence and molecular epidemiology of *bla*_*NDM−1*_among *Pseudomonas aeruginosa* isolates causing bloodstream infections in Egypt, while characterizing their antimicrobial susceptibility profiles, carbapenemase gene distribution, and biofilm-forming ability, and exploring their association with metallo-β-lactamase genes. Furthermore, the study elucidated the genomic context and resistome of *bla*_*NDM−1*_–producing isolates using whole-genome sequencing.

## Methods

### Antimicrobial susceptibility testing

In the period between February 2023 and December 2023, a total of 50 non-duplicate *P. aeruginosa* clinical isolates were gathered from blood samples of cancer patients admitted to the National Cancer Institute (NCI), Cairo University, Egypt. Isolates from other infection sites were not included to maintain a homogeneous sample source and focus specifically on bloodstream infections in this high-risk population. Samples were initially identified using standard microbiological techniques then further confirmed using the BD PHOENIX Automated Microbiology System (Becton-Dickinson Diagnostic Systems, Sparks, USA). *E. coli* ATCC 25,922 was used as the control strain [11].

Susceptibility testing and MICs determination were conducted and interpreted following the EUCAST 2022 guidelines [12]. Bacterial suspensions were adjusted to a 0.5 McFarland standard (~ 1 × 10⁸ CFU/mL), and inoculation was performed on Mueller–Hinton agar plates followed by incubation at 35 ± 2 °C for 16–18 h under aerobic conditions. The disc diffusion method using Oxoid discs (from Oxoid Ltd., Basin Stoke, Hants, England) was utilized for (imipenem, meropenem, ceftazidime, cefepime, piperacillin/tazobactam, amikacin, levofloxacin, and ciprofloxacin). Colistin MIC was assessed employing broth microdilution method using colistin sulphate (Sigma Aldrich, UK ), while E-test strips from bioMerieux (Marcy L’Etoile, France) were employed to determine the MIC of ceftolozone/tazobactam. Quality control for colistin broth microdilution was performed using *E. coli* ATCC 25,922 and *P. aeruginosa* ATCC 27,853 in accordance with EUCAST recommendations.

### Detection of carbapenemase genes

Multiplex PCR assays were initially designed to detect *bla*_*NDM*_-like genes, followed by confirmation of the *bla*_*NDM−1*_ variant using previously described primers. Two multiplex PCRs were performed to detect carbapenemases (*bla*_*KPC−like*_, *bla*_*NDM− like*_, *bla*_*IMP− like*_, *bla*_*VIM− like*_, *and bla*_*OXA−48− like*_). The first PCR targeted the detection of *bla*_*KPC*_, *bla*_*NDM−1*_, *and bla*_*OXA−48*_, while the second PCR focused on detecting *bla*_*IMP*_ *and bla*_*VIM*_, following established protocols and utilizing previously described primers [13, 14].

### Biofilm assay

The 96-well-plate optical method was performed as previously described [15]. Briefly, 10 µl of overnight cultures in 0.2% glucose trypticase soy broth (TSB) was adjusted to a 0.5 McFarland standard was added to 190 µl of TSB in three wells of a 96-well microtiter plate, then incubated at 37 °C for 24 h. Following incubation, the bacterial suspensions were removed, wells were washed with phosphate-buffered saline, air-dried, and stained with a crystal violet solution. After staining, the excess stain was washed off, and the wells were solubilized with acetic acid. Optical density was measured at 630 nm. Negative control wells without inoculum were included. Results were recorded as mean absorbance readings from triplicate wells. Biofilm producing isolates were categorized into weak, moderate or strong biofilm producers based on the methodology described by Sherif et al.

### Multi-locus sequence typing (MLST)

Ten isolates representing the *bla*_*NDM-1*_ producing isolates were subjected to MLST. These isolates were selected as a representative subset of *bla*_*NDM-1*_–producing isolates for molecular typing. Selection was based on their representation of different antimicrobial resistance profiles, biofilm formation capacities, and to include isolates from the predominant *bla*_*NDM-1*_–producing group, ensuring coverage of phenotypic diversity. PCR and sequencing of the 7 housekeeping genes (*acsA*,* aroE*,* guaA*,* mutL*,* nuoD*,* ppsA*,* and trpE*) were done [16]. Sequences of these genes were compared with the sequences submitted to the MLST database to determine the allelic numbers and sequence types.

### Whole genome sequencing (WGS) and analysis

Whole genome sequencing and analysis was used to detect the *bla*_*NDM− 1*_ genomic environment and resistome in *bla*_*NDM− 1*−_producing strains. Two *bla*_*NDM− 1*_ - producing isolates representing the identified sequence types (ST308 and ST773) were selected for whole-genome sequencing to investigate and compare the genomic context of resistance. However, the limited number of sequenced isolates may not fully capture the genomic diversity within these lineages. Nanopore^®^ long-read technology was conducted using MinION Flow Cell (R10.4.1) and MinION Mk1C device (Oxford Nanopore Technologies, UK) [17]. The resultant filtered reads underwent *de novo* assembly and polishing processes employing Flye [18] and Medaka tools https://github.com/nanoporetech/medaka, respectively. Identification of antimicrobial resistance genes (AMR) was accomplished through Amrfinderplus and CARD system https://card.mcmaster.ca/ [19, 20]. Analysis of the genetic environment of the flanking regions of the detected *bla*_*NDM*_ genes was done using ICEfinder https://bioinfo-mml.sjtu.edu.cn/ICEfinder/ICEfinder.html, Blast webtool https://blast.ncbi.nlm.gov/. Proksee https://proksee.ca/ was used for visualization while its incorporated tools like align hunter and mobile-OG was used to detect horizontal gene transfer regions and other mobile genetic elements respectively [21]. The resultant ICE consensuses were annotated using Bakta https://bakta.computational.bio/ annotation tool. Assemblies were deposited under the bioproject accession number PRJNA1131125. Integrative and conjugative elements (ICEs) were identified based on established bioinformatic criteria; no experimental conjugation or transfer assays were performed.

### Statistical analysis

All the statistical analyses were performed using Graphpad Prism version 8.0.2. Fisher’s exact test was implemented where categorical variables were compared, P value ≤ 0.05 was considered statistically significant.

## Results

### Antimicrobial susceptibility

A total of 50 *P. aeruginosa* isolates responsible for bloodstream infections among hospitalized cancer patients were confirmed, all exhibiting MDR/XDR phenotypes. The highest resistance prevalence was observed against ceftazidime, piperacillin/tazobactam, cefepime, and imipenem (≥ 75%). The lowest resistance rates were observed for ceftolozone/tazobactam, aztreonam, and colistin being only 22% (*n* = 10) for the later. Resistance to all the tested antipseudomonal drugs (ceftazidime, piperacillin/tazobactam, cefepime imipenem, meropenem, levofloxacin, ciprofloxacin, and aztreonam) were observed among 32% (*n* = 16) of the isolates. Among the 50 isolates, 80% (*n* = 40) were CRPA; resistant to imipenem and/or meropenem with higher susceptibility to meropenem over imipenem. Colistin susceptibility was the most abundant among carbapenem resistant (CR) isolates with 80% (*n* = 32) susceptibility. Aztreonam and ceftolozone/tazobactam displayed activity against 42% (*n* = 16) and 35% (*n* = 14) of the CR isolates respectively.

### Prevalence of carbapenemase genes

**Fig. 1:**
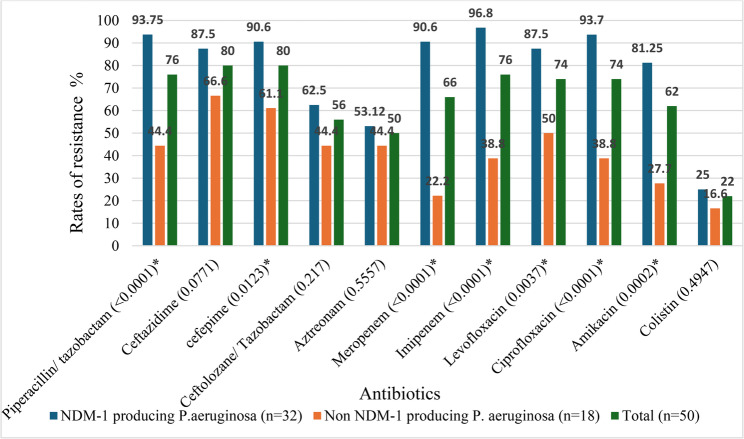
Antimicrobial resistance rates for the NDM-1–producing and –nonproducing *P. aeruginosa* isolates. * Denote significant difference between NDM-1–producing and –nonproducing *P. aeruginosa* isolates < 0.05

Detection of carbapenemase genes revealed that blaNDM−1 was the most abundant gene, with a prevalence of 64% (n = 32) among all isolates and 80% of CR isolates, while only 2 isolates (4%) harbored blaKPC. None of the other tested genes (blaOXA−48, blaVIM, blaIMP) were detected. All carbapenemase producers were resistant to meropenem and/or imipenem. As expected, blaNDM−1 carriage was associated with significantly higher resistance levels to carbapenem. In addition, high resistance levels toward other antibiotics were detected among the blaNDM−1 producing isolates, as shown in Fig. 1.

### Biofilm assay results

All isolates were biofilm producers, with 40% (*n* = 20) strong biofilm formers and 60% (*n* = 30) moderate or weak biofilm formers (Table 1). Seventeen (85%) of the strong biofilm producing isolates were blaNDM-1 producing. While a significant association between blaNDM-1 and strong biofilm formation (*P* = 0.0115) was observed, no multivariate analysis was performed to control for potential confounding factors, which may influence this relationship.

**Table 1:** Resistance patterns, MICs, carbapenemase genes and sequence types of the *P. aeruginosa* isolates in this study

Isolate number	Meropenem	Imipenem	Piperacillin/ tazobactam	Ceftazidime	Cefepime	Ceftolozane/ Tazobactam	Aztreonam	Levofloxacin	Ciprofloxacin	Amikacin	Colistin (MIC)	Carbapenemase genes	Sequence types (ST)	Biofilm formation ability
1	S	S	S	R	R	S	S	R	S	S	R(8)	(-ve)		Non-strong
2	S	S	R	R	S	R	S	S	S	S	S(4)	(-ve)		Non-strong
3	S	S	S	S	S	S	S	S	S	S	S(4)	(-ve)		Non-strong
4	R	S	R	R	R	R	S	R	S	S	S(2)	(-ve)		Strong
5	R	R	R	S	R	R	S	R	R	R	S(4)	NDM		Strong
6	S	S	S	R	S	S	S	S	S	S	S(4)	(-ve)		Non-strong
7	S	S	S	S	S	R	S	S	S	S	S(4)	(-ve)		Non-strong
8	S	S	S	R	S	S	S	S	S	S	S(4)	(-ve)		Non-strong
9	S	R	S	S	S	R	R	R	S	S	S(4)	(-ve)		Non-strong
10	R	R	R	R	R	R	R	R	R	R	S(4)	NDM		Non-strong
11	R	R	R	S	R	S	S	R	R	R	S(2)	NDM	773	Strong
12	R	R	R	R	R	R	R	R	R	R	S(4)	NDM		Strong
13	R	R	R	R	R	R	R	R	R	R	S(4)	NDM		Non-strong
14	R	R	R	R	R	S	S	R	R	R	S(4)	NDM		Strong
15	R	R	R	R	R	R	R	R	R	R	S(4)	NDM	308	Non-strong
16	R	R	R	S	S	R	S	R	R	R	S(4)	NDM		Non-strong
17	S	R	S	S	R	S	S	S	S	S	S(4)	(-ve)		Non-strong
18	R	R	R	R	R	R	S	R	R	R	S(4)	NDM		Strong
19	R	R	R	R	R	R	S	R	R	R	R(8)	NDM	773	Strong
20	S	R	S	R	R	R	R	R	R	S	R(8)	NDM		Strong
21	R	R	R	R	R	R	R	S	R	S	S(4)	KPC		Non-strong
22	S	R	R	S	R	R	R	R	R	S	S(4)	(-ve)		Strong
23	R	R	R	R	R	S	S	R	R	R	R(8)	NDM		Non-strong
24	R	R	R	R	R	S	R	R	R	R	R(8)	NDM	773	Strong
25	R	R	R	R	R	R	R	S	R	R	S(4)	NDM		Strong
26	R	R	R	R	R	R	S	R	R	S	S(4)	NDM		Strong
27	R	R	S	R	R	R	R	R	R	R	S(4)	NDM		Strong
28	S	R	R	S	S	S	S	S	S	S	S(4)	NDM		Strong
29	R	R	R	R	R	R	S	R	R	S	S(4)	NDM		Strong
30	R	R	R	R	R	S	R	R	R	R	S(4)	NDM		Non-strong
31	S	R	R	R	S	S	R	S	S	S	S(2)	NDM		Non-strong
32	R	R	R	R	R	R	R	R	R	R	R(8)	NDM	773	Strong
33	R	R	R	R	R	R	R	R	R	S	S(2)	NDM		Non-strong
34	S	R	R	R	R	R	R	S	R	S	S(4)	(-ve)		Non-strong
35	S	S	R	R	R	S	R	R	R	R	R(16)	(-ve)		Non-strong
36	R	R	R	R	R	R	R	R	R	R	S(4)	NDM		Non-strong
37	S	S	S	R	R	S	S	R	S	R	R(32)	(-ve)		Non-strong
38	R	S	R	R	R	S	R	R	R	R	R(32)	NDM		Non-strong
39	R	R	R	R	R	S	S	R	R	R	S(4)	NDM	308	Non-strong
40	R	R	R	R	R	S	S	R	R	R	S(4)	NDM		Non-strong
41	S	S	S	S	S	S	S	S	S	S	S(2)	(-ve)		Strong
42	R	R	R	R	R	R	S	R	R	R	S(4)	NDM	308	Strong
43	R	R	R	R	R	R	S	R	R	R	R(32)	NDM		Non-strong
44	R	R	R	R	R	R	R	S	R	R	R(32)	NDM	308	Strong
45	S	S	S	R	R	S	R	R	R	R	S(2)	(-ve)		Non-strong
46	R	R	R	R	R	S	S	R	R	R	S(4)	NDM	308	Non-strong
47	R	R	R	R	R	R	R	R	R	R	S(4)	NDM		Strong
48	R	R	R	R	R	S	R	R	R	R	S(4)	NDM	308	Non-strong
49	R	R	R	R	R	R	R	R	R	R	S(4)	KPC		Non-strong
50	R	R	R	R	R	S	R	R	R	R	S(4)	(-ve)		Non-strong

All isolates were biofilm producers, with 40% (*n* = 20) strong biofilm formers and 60% (*n* = 30) moderate or weak biofilm formers (Table 1). Seventeen (85%) of the strong biofilm producing isolates were *bla*_*NDM-1*_ producing. While a significant association between *bla*_*NDM-1*_ and strong biofilm formation (*P* = 0.0115) was observed, no multivariate analysis was performed to control for potential confounding factors, which may influence this relationship.

### MLST

Ten *bla*_*NDM−1*_–producing isolates were selected for MLST analysis based on their representation of diverse antimicrobial resistance profiles and biofilm-forming capacities, as well as to include isolates reflecting the predominant phenotypic patterns observed in the study. This selection approach was adopted to capture representative diversity; however, it may not fully reflect the entire clonal distribution of bla_*NDM−1*_–producing isolates.

Among these ten bla_*NDM−1*_–producing isolates, only two sequence types were identified, with six isolates assigned to ST308 and four isolates assigned to ST773. Notably, one ST773 isolate exhibited resistance to all tested antimicrobial agents, including colistin. These findings are based on a representative subset and may not fully reflect the overall diversity of all *bla*_*NDM−1*_–producing isolates. The detailed antibiogram, colistin MIC values, presence of carbapenemase genes, distribution of sequence types among the sequenced isolates, and biofilm formation intensity are presented in Table 1.

### Resistome analysis

A WGS-based resistome analysis revealed the presence of antimicrobial resistance genes (ARG) conferring resistance to a wide range of antibiotics in both STs. In ST308, genes for resistance to aminoglycosides (*aph(3’’)-Ib*,* aph(3’)-IIb*,* aph(6)-ld*,* aadA11*,* aac(6’)-Ib*,* aac(6’)-II*,* aac(3)-Id and rmtF2*), beta- lactams (*bla*_*NDM−1*_,*bla*_*PAC−1*_, *bla*_*PDC−7*,_ *bla*_*PDC−19a*_, *bla*_*OXA−10*_, *and bla*_*OXA−488*_), chloramphenicol (*catB7*), quinolones (*crpP and qnrVC1)*, fosfomycin (*fosA*), sulfonamides (*sul1 and sul2*), trimethoprim (*dfrB5*), macrolides (*msrE*), and the biocide quaternary ammonium *(qacE*) were detected. ST773 harbored genes mediating resistance to aminoglycosides (*aadA11*,* aph(3’)-IIb*,* acc(3)-ld*,* and rmtB4*), β-lactams (*bla*_*NDM−1*,_ *bla*_*PDC−16*_, *bla*_*PDC−2*_ *and bla*_*OXA−395*_), chloramphenicol and florfenicol (*catB7* and *floR*), quinolones (*crpP* and *qnrVC1)*, fosfomycin (*fosA*), sulfonamides (*sul1*), tetracycline *tet(G)*, bleomycin (*ble*), and the biocide quaternary ammonium (*qacE*).

### Genomic environment of *bla*_*NDM−1*_

The *bla*_*NDM−1*_ gene in both sequence types was integrated into the chromosome by an integrative conjugative element (ICE) likely involving IS91 transposase sequences, as revealed by analysis using ICEfinder. The size of the ICE in ST308 was approximately 248 Kb. The *bla*_*NDM−1*_ gene was part of a cassette that also contained two other resistance genes, *floR* and *msrE*, in the following arrangement: (*floR →* DUF3366 *→* IS91 transposase *→ msrE →* IS91 transposase sequence *→bla*_*NDM−1*_ *→*IS91 transposase sequence). BLASTN analysis showed that this genetic arrangement had been described previously within ICE Tn43716385 [22]. Although comparable genetic arrangements have been described previously, the complete ICE sequence remains poorly characterized in the literature.

**Fig. 2:**
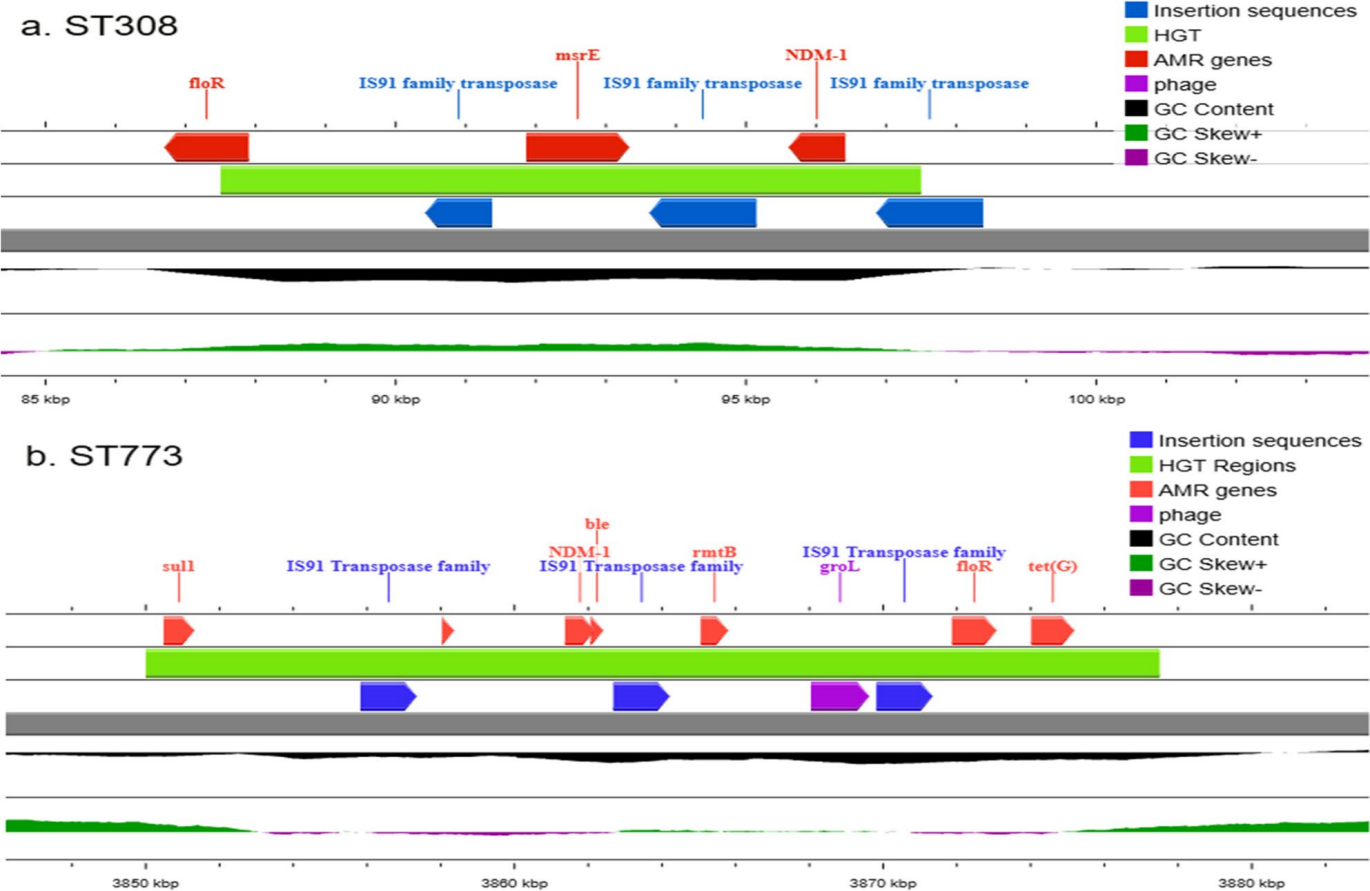
Genetic environment of *bla*_NDM−1_ gene in carbapenem resistance *P. aeruginosa* ST308 (**a**) and ST773 (**b**)

The ICE size in ST773 was about 117 Kb. The whole cargo was part of putative mobile element also carrying *sul1*,* Acc(3)*, *rtmB*,* floR*, ble, and *tet(G)* in the following arrangement (*sul1→ TniQ → TniB → TniA → IS91* transposase genes *→ Acc(3)* *→ IS91* transposase genes *→ blaNDM-1 → ble → IS91* transposase genes *→ rmtB →* metallophosphoestrase encoding gene *→ Tgt → Grol → IS91* transposase genes *→ floR2 → tet(G)*). BLASTN of the arrangement in ST773 revealed 100% similarity with the previously reported *P. aeruginosa* clc-like ICE mobile element named ICE6660 (GenBank: MK497171.1) [23] (Fig. 2).

## Discussion

The overuse of carbapenem is a worldwide concern as it contributes to the emergence of multidrug-resistant pathogens [24]. This study demonstrated a high level of carbapenem resistance (80%, *n* = 40) among *P. aeruginosa* isolates causing blood stream infections in immunocompromised patients.

This observation may be partially explained by previous reports suggesting that overuse of carbapenems as empirical therapy resistance may contribute to resistance development among *P. aeruginosa* clinical isolates [25, 26]. MBLs are one of the key mechanisms governing the carbapenem resistance spread among Gram negative bacteria within clinical settings [27]. Alarmingly, in the present study, the overall prevalence of the MBLs *bla*_*NDM−1*_ gene (*n* = 32) was 64% and 80% among CRPA. Although carbapenemase mediated CR among *P. aeruginosa* was often derived by other MBLs such as *bla*_*VIM*_ and *bla*_*IMP*_ [4, 6]. Our results together with other reports suggest a possible shift in the molecular epidemiology of CRPA in high-risk patients characterized increasing detection of *bla*_*NDM−1*_ [2, 3, 6, 7, 26]. This shift has been attributed to the endemicity of blaNDM-1 in healthcare settings and its association with mobile genetic elements that facilitate horizontal gene transfer among Gram-negative pathogens, all of which have consistently shown a high prevalence of this gene [4, 5]. Notably, this study showed a low prevalence of *bla*_KPC_ among CRPA. Although it is primarily associated with *K. pneumoniae*, we emphasize the need for proactive measures to limit its transmission among *P. aeruginosa* isolates, as sporadic cases have been reported worldwide [28].

There are scarce therapeutic options available for patients with infections caused by *P. aeruginosa* carrying the *bla*_NDM−1_ gene [3]. Although amikacin and fluoroquinolones are considered useful antipseudomonal therapy [29], their resistance rates among *bla*_*NDM−1*_ producing isolates revealed by this study are worrisome (*n* = 26, 81.25%; *n* = 28, 87.25%). Particularly when considering the resistance rates of ceftolozane/tazobactam (*n* = 31, 62%) and aztreonam (*n* = 26, 53.2%), which raise alarm despite their relatively lower resistance rates. Though aztreonam is not hydrolyzed by MBL, our isolates showed high non susceptibility rates [29]. Our findings come hand to hand with other reports that confirmed similar rates of aztreonam non susceptibility toward CRPA [29]. This study revealed that colistin remains one of the limited therapeutic options; however, its use is constrained by nephrotoxicity and limitations in susceptibility testing.

In the current study, all *P. aeruginosa* isolates were biofilm producers with variant intensities. The formation of strong biofilms was significantly associated with the production of *bla*_*NDM−1*_ gene, a finding supported by other studies that ascribed this to the interplay between carbapenemases and some regulatory pathways controlling biofilm formation, which may result in upregulation of key biofilm-associated genes [13, 14]. This association is correlative and does not imply direct causation, as biofilm formation is influenced by multiple genetic and regulatory factors. In addition, the environmental pressures generated by carbapenemases could possibly endorse the development of biofilms as a survival tactic in CRPA [10]. While this connection has been proposed, it’s important to address that a portion of non *bla*_*NDM−1*_-producing isolates showed a strong biofilm production and vice versa. This suggests that the production of the *bla*_*NDM−1*_ is not solely responsible for biofilm formation; rather, it is likely influenced by multiple factors.

Worldwide surveillance has linked the dissemination of *bla*_*NDM−1*_ among *P. aeruginosa* to a specific genetic lineage [30]. Herein, only two clones (ST773 and ST308) were associated with the dissemination of this gene in our hospital, both clones were reported to harbor *bla*_*NDM−1*_ before [8, 22]. WGS analysis of two isolates, each representing one of the detected STs, revealed a vast arsenal of ARGs conferring resistance to nearly all classes of antimicrobial agents in both clones. The co-presence of genes conferring resistance toward almost all antibiotic such as beta lactams (at least one *bla*_*OXA*_ and *bla*_*PDC*_ variant), aminoglycosides (*aph(3’)-IIb*,* aadA11*,* aac(3)-Id and rmt)*, quinolones (*crpP and qnrVC1)*, fosfomycin (*fosA*), sulfonamides (*sul1) and* chloramphenicol (*catB7*) was detected in both isolates. Dissemination of both clones harboring these genes within the facility may explain the significantly higher resistance levels among *bla*_*NDM−1*_ carrying isolates than non-*bla*_*NDM−1*_ carrying isolates. For instance, resistance levels were significantly higher in *bla*_*NDM−1*_ carrying isolates for piperacillin/tazobactam (*n* = 30, 93.75%. *P* < 0.0001), amikacin (*n* = 26, 81.25%, *P* = 0.0002), levofloxacin (*n* = 28, 87.25%, *P* = 0.0037), ciprofloxacin (*n* = 30, 93.75, *P* < 0.0001). Predictably, meropenem and imipenem resistance was significantly higher in *bla*_*NDM−1*_ carrying isolates than non-*bla*_*NDM−1*_ carrying isolates (*n* = 29, 90.625%, *P* < 0.0001; *n* = 31, 96.875%, *P* < 0.0001). The dissemination of both clones should be carefully monitored and controlled, as it poses a significant challenge to the clinical management of infections. This complex array of resistance determinants not only limits the efficacy of available antibiotics but also poses a serious threat to public health due to the potential for treatment failures.

In this study, WGS analysis revealed that transposons belonging to IS91family delegated the incorporation of *bla*_*NDM−1*_ gene into an ICE in both sequence types. The involvement of IS91-like elements has been predominantly associated with the mobilization of antimicrobial resistance genes, including carbapenemases [31, 32].

Our predominant clone, *P. aeruginosa* ST308 producing *bla*_*NDM−1*_, is reported for the first time in Egypt by our group [8], despite being recognized as a globally disseminated high-risk epidemic clone [7, 27, 32]. The genetic context of *bla*_*NDM−1*_ in *P. aeruginosa* ST308 indicates that *bla*_*NDM−1*_ is chromosomally integrated into an integrative and conjugative element within a single cassette alongside two other resistance genes, *floR* and *msrE*. This arrangement was described before within ICE Tn43716385 [33, 34]. Moreover, this ICE may facilitate the transferability multiple resistance as it confers a triad resistance witnessed by harboring resistance genes against macrolides, florfenicol as well as carbapenems. The detection of this clone among Egyptian healthcare settings is concerning due to its inter-clonal diversity that chaperoned the evolution of multiple lineages that occupy various niches within the complex ecosystem of a hospital, thereby facilitating its epidemic behavior [35]. Considering this, we highlight the need for the urgent need to continued surveillance of ST308 due to the challenge it imposes in hospital environment leading to substantial outbreaks.

ST773 has been described as a high-risk clone disseminated worldwide but it was associated with *bla*_*NDM−1*_ only since 2018 [36]. This work revealed the genetic environment of ST773 which is closely related to previously reported ST773-*P. aeruginosa* isolates in which an ICE6660-like, carrying *sul1*,* Acc(3)*, *rtmB*,* floR*, *ble*, and *tet(G)* was reported [7, 21–23, 34]. The co-presence of *rmtB* and *acc3* alongside *bla*_*NDM−1*_ is particularly concerning, as the transmission of such an integrative and conjugative element (ICE) significantly limits therapeutic options. This co-resistance mechanism compromises the efficacy of two significant antipseudomonal drug classes, aminoglycosides and carbapenems.

In both clones, the presence of multiple resistance genes (ST308: *bla*_*NDM−1*_, *floR* and *msrE; ST773: sul1*,* acc(3)*, *rtmB*,* floR*, ble, and *tet(G))* on a platform of genomic plasticity like the ICE warrants special attention. More worrying is the presence of IS91 transposases which are usually associated with the mobility of antimicrobial resistance genes. Both events underline the potential of the ICE, suggesting the strain may have the ability to acquire additional resistance determinants. This ongoing acquisition poses a persistent threat to antimicrobial efficacy within these clones and raises the possibility of horizontal gene transfer to other species [7, 29, 34].

Tokuda et al., stated that ICEs are more frequently transferred among diverse species than conjugative plasmids, implying that ICEs might have broader host ranges [37]. Given the endemicity of *bla*_*NDM−1*_ among gram negative bacteria in Egyptian hospitals and its association within ICEs, we hypothesize that this may reflect a broader trend in the molecular epidemiology of carbapenem resistance in CRPA is driven by *bla*_*NDM−1*_ rather than other previously dominant metallo-β-lactamases (MBLs) like *bla*_*VIM*_ and *bla*_*IMP*_, whose transmission primarily occurs through plasmids.

### Limitation

This study has some limitations. First, it was based on a relatively small sample size of 50 bloodstream isolates, which may not fully capture the diversity of carbapenem-resistant *P. aeruginosa* circulating in Egypt. Second, the isolates were obtained from a single oncology center, which limits the findings to other geographic regions and healthcare settings. Finally, only two isolates were subjected to whole-genome sequencing, providing valuable insights into resistance mechanisms; however, this limited number restricts the generalizability of the genomic findings and does not fully capture the diversity and variability of resistance mechanisms within the studied population. Future multicenter studies with larger numbers of isolates and expanded sequencing are needed to validate and extend these findings.

## Conclusion

The current study provides insights into *Pseudomonas aeruginosa* ST308 carrying *bla*_*NDM−1*,_ reported for the first time in Egypt, alongside ST773, both of which represent high-risk multidrug-resistant clones. The dissemination of *bla*_*NDM−1*_ in Egyptian healthcare settings appears to be driven by a combination of horizontal gene transfer, mediated through integrative and conjugative elements (ICEs) with IS91 transposases, and clonal spread of these resistant lineages. This may reflect a broader epidemiological drift in carbapenem-resistant *P. aeruginosa* from other MβLs towards *bla*_*NDM−1*_, provoking particular concern due to the frequent co-occurrence of multiple antimicrobial resistance determinants that drastically restrict treatment options.

Clinically, these findings underline the urgent need to minimize the risk of *bla*_*NDM−1*_ **-**producing *P. aeruginosa* infections and their spread, especially in immunocompromised cancer patients who are highly susceptible to bloodstream infections and poor outcomes. The convergence of extensive resistance profiles with highly mobile genetic platforms highlights the potential for rapid dissemination in hospital environments, necessitating strict infection prevention and control measures, reinforced antimicrobial stewardship, and the implementation of genomic surveillance programs.

## Data Availability

The datasets used during the current study are provided within the manuscript. The nucleotide sequences of the whole genomes can be accessed in the full-length genomes of the isolates deposited in the GenBank database under the Bio Project accession number PRJNA1131125, with GenBank accession numbers SAMN42266028 (ST773) and SAMN42266031 (ST308).
